# Association between electroconvulsive therapy and depressive disorder from 2012 to 2021: Bibliometric analysis and global trends

**DOI:** 10.3389/fnhum.2022.1044917

**Published:** 2022-11-15

**Authors:** Haipeng Cai, Ruonan Du, Kebing Yang, Wei Li, Zhiren Wang

**Affiliations:** Beijing Huilongguan Hospital, Peking University Huilongguan Clinical Medical School, Beijing, China

**Keywords:** electroconvulsive therapy, depressive disorder, CiteSpace, bibliometrics analysis, global trends

## Abstract

**Background:**

Depressive disorder is a chronic mental illness that is vulnerable to relapse, imposes a huge economic burden on society and patients, and is a major global public health problem. Depressive disorders are characterized by depressed mood, decreased energy and interest, and suicidal ideation and behavior in severe cases. They can be treated through pharmacotherapy and psychotherapy or physical treatments such as electroconvulsive therapy (ECT). In patients with suicidal ideation, behavior, or refractory depressive disorder ECT has a faster onset of action and better efficacy than pharmacotherapy. This study used bibliometric and visual analyses to map the current state of global research on ECT for depressive disorder and to predict future research trends in this area.

**Materials and methods:**

A literature search was performed for studies on ECT and depressive disorder in the Web of Science Core Collection (WoSCC) database. All studies considered for this paper were published between 2012 and 2021. Bibliometric and co-occurrence analyses were performed using the CiteSpace software.

**Results:**

In total, 2,184 publications were retrieved. The number of publications on ECT and depressive disorder have been increasing since 2012, with China being a emerging hub with a growing influence in the field. Zafiris J. Daskalakis is the top author in terms of number of publications, and *The Journal of ECT* is not only the most published journal but also the most co-cited journal in the field. Co-occurrence analysis showed that electroconvulsive therapy, treatment-resistant depression, bipolar disorder, hippocampus, efficacy, and electrode placement are current research hotspots. Molecular biomarkers, neuroimaging predictors, and late-life depression will become research hotspots in the future.

**Conclusion:**

Our analysis made it possible to observe an important growth of the field since 2012, to identify key scientific actors in this growth and to predict hot topics for future research.

## Introduction

Depressive disorder is a chronic, easily relapsing mental illness with depressed mood, decreased energy, and diminished interest as its core symptoms. It frequently presents, in addition, problems with appetite, sleep, memory, and concentration, or psychotic symptoms, such as hallucinations and delusions. In severe cases, it can also involve suicidal ideation and behavior. The World Health Organization has reported that more than 264 million people worldwide have depressive disorders (World Health Organization [WHO], 2020). Epidemiological surveys from China show that depressive disorder is the most prevalent mood disorder, with a lifetime prevalence of 3.4% (Huang et al., 2019). As the number of people with this disorder is rising annually and is prone to relapse, it imposes a huge economic burden on society and families.

Treatment for depressive disorders is usually based on medication, psychotherapy, or a combination of both, but approximately 30% of patients with major depressive disorder do not respond well to these treatments (Rush et al., 2006). Electroconvulsive therapy (ECT) is a physical treatment method for psychiatric disorders that stimulates widespread electrical discharges in the patient’s cerebral cortex through a certain amount of electric current. These produce a series of neurophysiological and biochemical responses, thus relieving clinical symptoms. Guidelines (Jaffe, 2002; National Institute for Clinical Excellence [NICE], 2003; Ravindran et al., 2009) for specific indications for ECT vary depending on the country; however, ECT efficacy for some diseases seem to be consistently accepted across countries. ECT is generally considered more effective, fast-acting, and safe for patients with major depressive disorder (especially when psychotic symptoms are present or when drug resistance and/or suicidal ideation are present), catatonic symptoms, and major manic episodes (Tor et al., 2021).

Currently, global trends in ECT for depressive disorders are unclear. Bibliometrics is a comprehensive analysis method, which can fill this gap by analyzing publications to qualitatively and quantitatively assess the contributions of countries, institutions, and authors in the field and predict future research trends (Chen et al., 2014; Wang et al., 2021). This study aims at investigating global research trends in ECT for depressive disorders over the past 10 years and at predicting future research hotspots within the field through bibliometric and visual analysis.

## Materials and methods

### Data sources

The data for this study were retrieved from WoSCC, a wide and high quality database for bibliometric data. We conducted a search of the WoSCC database on 14 June 2022.

**FIGURE 1:**
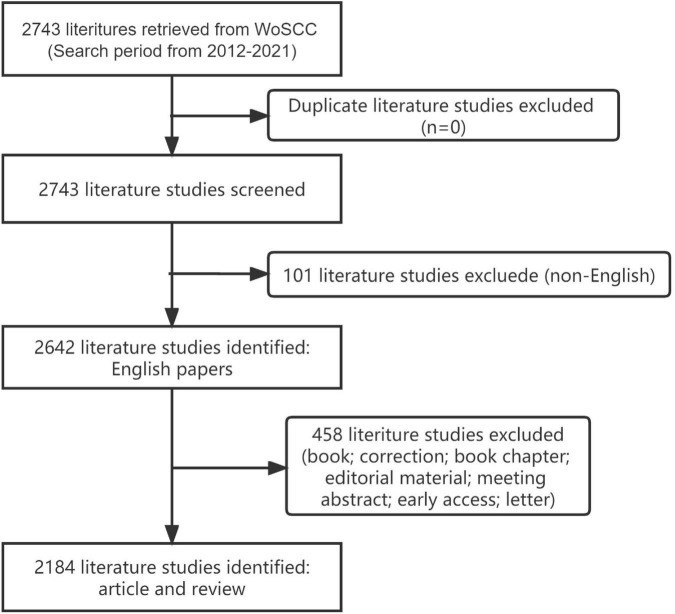
Flow chart for including and excluding literature studies.

The search strategy used in our study was TS = electroconvulsive therapy AND (TS = depressive disorder OR depression). The search time ranged from 2012 to 2021. The language used for the search was English. We excluded “meeting abstract“, “editorial material”, “letter”, and “early access”. Given our exclusion criteria, the remaining 2184 paper were mostly composed of original articles and meta-analyses (Figure 1).

### Tools and data analysis

CiteSpace is a Java program, developed by Professor Chao-Mei Chen at Drexel University, that visualizes literature data from various databases. The software has been used to analyze trends in the evolution of disciplinary research frontiers and internal links between different research frontiers. Collaborative networks can be used to identify core countries/regions, institutions, authors, and their collaborations. Co-occurring keywords can reveal both the starting point of a subfield of research and current research hotspots. In addition, detecting bursts can detect research trends more clearly.

In this study, we used WoSCC to analyze publication output, scientific subject category networks, year of publication, and other data such as impact factors. As mentioned earlier, relevant literature was searched using keywords and unqualified literature was filtered. The selected literature information was then saved and imported into CiteSpace v5.8 R3.

The specific parameters were set as follows. Time slice: January 2012–December 2021; term source: title, abstract, author keywords, and keywords; node type: author, institution, country, keyword, and journal; link strength: cosine; selection criteria: Top *N* = 50; Pruning: MST.

Centrality is a key indicator of the importance of the keywords analyzed in the CiteSpace output. If the centrality of a node exceeds 0.1, it means that the node is a central node, which is more important and has more influence in the study. In the generated visualization image, the node size represents the frequency of keyword co-occurrences; the larger the node, the higher the frequency. The color of the center of the node represents the time of keyword appearance; the closer the color is, the earlier the keyword first appears. The red areas in the nodes indicate the occurrence of a citation or frequency burst. Burst detection can be used to analyze the current status of research and hotspots, suggesting future research trends in the field.

## Results

### Distribution of annual publication

**FIGURE 2:**
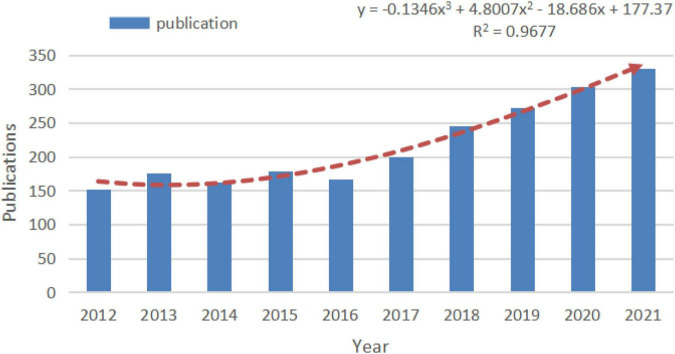
The number of publications in recent 10 years.

Changes in the quantitative distribution of publications are important indicators that can directly reflect the trends in the field. The annual publications on depressive disorder and ECT in the last 10 years included 2184 relevant articles, and the overall trend showed an increase of publications on depressive disorder and ECT each year. This indicates ECT or depressive disorder as a hot topic of research (Figure 2).

### Analysis of scientific collaboration network

**FIGURE 3:**
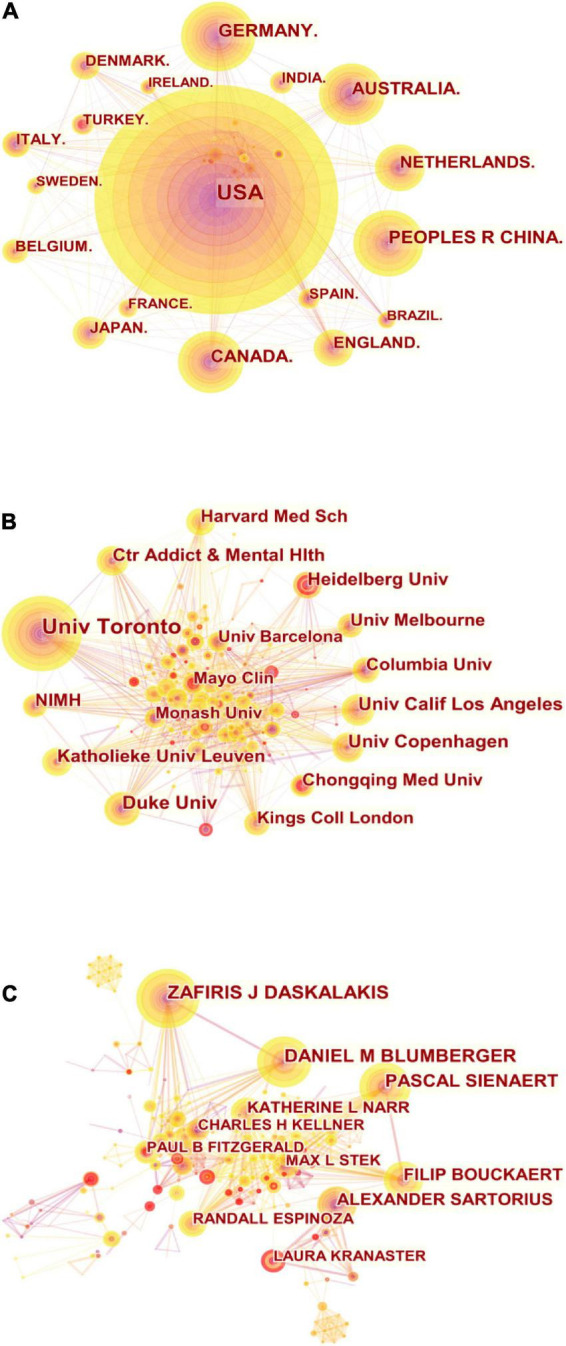
**(A)** Collaboration among countries/regions. **(B)** Collaboration among institutions. **(C)** Collaboration among authors.

A total of 73 countries/regions have publications in the field of ECT and depressive disorder. The top 10 countries/regions were ranked by publication and centrality (Table 1). The top five countries/regions by number of publications were the USA, Germany, China, Australia, and Canada, and the top five countries/regions by centrality were the U.S., Canada, the U.K., Australia, and Italy. Pearson’s correlation analysis revealed a significant correlation between publications and centrality at the country/region level (*r* = 0.890, *p* < 0.001). The comprehensive analysis of publications and centrality shows that the United States made the largest contribution to this field, with 671 publications and a centrality of 0.29, and Canada (publications: 187, centrality: 0.14) also has a large influence on this field. The network map of cooperation between countries/regions is shown in Figure 3A, with 80 nodes and 444 linked lines. The nodes and the linked lines between the nodes represent the countries/regions and their cooperation relationships, respectively. The larger the node, the more published content. The change in color indicates different publication dates, with sections closer to purple indicating earlier publication and sections closer to yellow indicating that the article was published more recently. It is worth noting that China ranks third in terms of the number of articles published and has seen a surge in the number of articles published in recent years, indicating that China has been contributing more to the field.

**TABLE 1:** Top 10 countries/regions, institutions, and authors according to publications and centrality.

Items	Publications			Centrality		
		
	Ranking	Name	Number	Ranking	Name	Number
Country/Region	1	USA	687	1	USA	0.29
	2	Germany	210	2	Canada	0.14
	3	China	203	3	England	0.14
	4	Australia	190	4	Australia	0.08
	5	Canada	187	5	Italy	0.08
	6	Netherlands	143	6	Spain	0.08
	7	England	113	7	France	0.06
	8	Japan	98	8	Germany	0.05
	9	Denmark	86	9	China	0.05
	10	Italy	82	10	Brazil	0.04
Institution	1	University of Toronto	104	1	Columbia University	0.17
	2	Duke University	51	2	University of Toronto	0.15
	3	University of California	47	3	Harvard Medical School	0.15
	4	University of Copenhagen	45	4	King’s College London	0.14
	5	Center Addict & Mental Health	44	5	The University of Melbourne	0.11
	6	Katholieke University Leuven	42	6	University of California	0.07
	7	Heidelberg University	41	7	Heidelberg University	0.06
	8	Columbia University	40	8	Augusta University	0.06
	9	Harvard Medical School	40	9	Yale University	0.06
	10	Chongqing Med University	37	10	Duke University	0.05
Author	1	Zafiris J Daskalakis	54	1	Georgios Petrides	0.15
	2	Daniel M Blumberger	47	2	Harold A Sackeim	0.09
	3	Pascal Sienaert	44	3	Colleen Loo	0.08
	4	Alexander Sartorius	35	4	Udo Dannlowski	0.08
	5	Filip Bouckaert	34	5	Hamish Mcallister Williams	0.06
	6	Katherine L Narr	29	6	Shawn M Mcclintock	0.05
	7	Declan M Mcloughin	28	7	Sarah H Lisanby	0.05
	8	Randall Espinoza	24	8	Pascal Sienaer	0.05
	9	Max L Stek	23	9	Andre F Carvalho	0.04
	10	Paul B Fitzgerald	22	10	Daniel J Smith	0.04

A total of 351 institutions have published in this area over the last 15 years. Table 1 lists the top ten institutions based on their publications and centrality. The top five institutions that have contributed the most to this field are the University of Toronto, Duke University, University of California, University of Copenhagen, and the Center for Addiction and Mental Health. The top five institutions in terms of centrality are Columbia University, the University of Toronto, Harvard Medical School, King’s College London, and the University of Melbourne. A significant correlation between publications and centrality was observed at the institutional level (Pearson’s *r* = 0.761, *p* < 0.001). According to the analysis of publications and centrality, the University of Toronto was overwhelmingly dominant in terms of number of publications, with 104 publications, and its centrality was 0.15. Columbia University (publications: 40, centrality: 0.17) also played a significant role in this area. There were 351 nodes and 1442 linked lines in the collaborative relationships between institutions (Figure 3B). The density of the institutional collaboration network is 0.0235. As the figure shows, there is relatively close collaboration between institutions.

The top ten authors are listed in Table 1 based on publication and centrality, with three of them having published more than 40 articles in the field as core authors: Zafiris J. Daskalakis, Daniel M. Blumberger, Pascal Sienaert. Pearson’s correlation analysis revealed a possible correlation between author-level publications and centrality (*r* = 0.447, *p* < 0.001). Furthermore, Figure 3C shows the collaboration network among authors, which contains 452 nodes and 1315 links with a density of 0.0129. This suggests that there is collaboration among top researchers in the field, and that some might have formed actual research teams.

### Analysis of journals and co-cited journals

Articles on the application of ECT to depressive disorder have been published in 536 journals. The top 10 journals and co-cited journals were mainly in the fields of psychiatry, brain science, and behavioral medicine (Table 2). The most published journal is *The Journal of ECT*, followed by the *Journal of Affective Disorders* and *Biological Psychiatry*. The top 10 journals have impact factors above 3.00, with five journals having impact factors above 5.00. Co-cited journals are other journals that are co-cited by researchers. The analysis of journal co-citations shows the contribution of each journal to the field. The top three journals based on co-citation counts were *The Journal of ECT* (1535), *the American Journal of Psychiatry* (1480), and *the Journal of Affective Disorders* (1409).

**TABLE 2:** Top 10 journals and co-cited journals.

Items	Ranking	Name	Counts	IF (2021)
Journal	1	Journal of ECT	372	3.635
	2	Journal of Affective Disorders	139	4.839
	3	Biological Psychiatry	108	13.382
	4	Brain Stimulation	64	8.955
	5	European Neuropsychopharmacology	56	4.600
	6	Neuropsychopharmacology	52	7.853
	7	Clinical Neurophysiology	51	3.708
	8	Psychiatry Research	50	3.222
	9	European Psyhiatry	47	5.361
	10	Acta Psychiatrica Scanidinavica	42	6.392
Co-cited journals	1	Journal of ECT	1535	3.635
	2	American Journal of Psychiatry	1480	18.112
	3	Journal of Affective Disorders	1409	4.839
	4	Biological Psychiatry	1403	13.382
	5	Journal of Clinical Psychiatry	1193	4.384
	6	Archives of General	1141	NA
	7	British Journal of Psychiatry	1090	9.319
	8	Neuropsychopharmacology	966	7.853
	9	Lancet	792	79.321
	10	Acta Psychiatrica Scanidinavica	785	6.392

### Analysis of co-cited references

Table 3 lists the details of the top ten highly cited articles. An article published by Joshi et al. (2016) was cited 89 times and had a large impact in the field. Their study showed that ECT-induced neuroplasticity in the hippocampus and amygdala is associated with improved clinical responses. The second most cited study was a meta-analysis by Haq et al. (2015). Their results showed that the shorter the duration of depressive episodes, the better the treatment response to ECT. In addition, medication failure was a significant predictor of adverse response to ECT, which is useful for predicting the treatment response to ECT in depressive disorder patients in the clinical setting. However, we need to explore more reliable biological predictors. The third most cited article was published in *JAMA Psychiatry* by Redlich et al. (2016), who attempted to predict patients’ treatment response to ECT from structural MRI results. Their results showed a positive correlation between pretreatment subgenual cingulate volume and individual ECT response. In addition, there was an increase in hippocampal volume in patients treated with ECT, whereas this change was not observed in the MRI of patients who only received medication. There are clinical difficulties in predicting an individual patient’s response to ECT treatment. The aforementioned literature explores the factors associated with treatment response to ECT. The results suggest that routine structural MRI evaluation before ECT can help predict the individual treatment effect of ECT.

**TABLE 3:** Top 10 highly cited references.

Ranking	Cited by	References	Title (publication year)	Journal (IF2021)
1	89	Joshi et al., 2016	Structural Plasticity of the Hippocampus and Amygdala Induced by Electroconvulsive Therapy in Major Depression.(2016)	Biological Psychiatry (13.382)
2	80	Haq et al., 2015	Response of depression to electroconvulsive therapy: a meta-analysis of clinical predictors.(2015)	Journal of Clinical Psychiatry (4.384)
3	70	Redlich et al., 2016	Prediction of Individual Response to Electroconvulsive Therapy *via* Machine Learning on Structural Magnetic Resonance Imaging Data.(2016)	JAMA Psychiatry (21.596)
4	70	van Diermen et al., 2018	Prediction of electroconvulsive therapy response and remission in major depression: meta-analysis.(2018)	British Journal of Psychiatry (9.319)
5	65	Kellner et al., 2016b	Right Unilateral Ultrabrief Pulse ECT in Geriatric Depression: Phase 1 of the PRIDE Study.(2016)	American Journal of Psychiatry (18.112)
6	62	Semkovska et al., 2016	Bitemporal Versus High-Dose Unilateral Twice-Weekly Electroconvulsive Therapy for Depression (EFFECT-Dep): A Pragmatic, Randomized, Non-Inferiority Trial.(2015)	American Journal of Psychiatry (18.112)
7	57	Abbott et al., 2014	Hippocampal structural and functional changes associated with electroconvulsive therapy response.(2014)	Translational Psychiatry (6.222)
8	56	Oltedal et al., 2018	Volume of the Human Hippocampus and Clinical Response Following Electroconvulsive Therapy.(2018)	Biological Psychiatry (13.382)
9	55	Dukart et al., 2014	Electroconvulsive therapy-induced brain plasticity determines therapeutic outcome in mood disorders.(2014)	PNAS (11.205)
10	54	Kellner et al., 2016a	A Novel Strategy for Continuation ECT in Geriatric Depression: Phase 2 of the PRIDE Study.(2016)	American Journal of Psychiatry (18.112)

### Analysis of co-occurring keywords

CiteSpace can reveal the hot topics of the study by clustering analysis of co-occurring keywords. There were 501 co-occurring keywords in this study: the top ten keywords are listed in Table 4, according to their frequency. The top two most frequent keywords were “electroconvulsive therapy” and “major depression”, which were the main themes of this study, followed by “efficacy,” “disorder,” “depression,” “meta analysis,” “treatment resistant depression,” “double blind,” “bipolar disorder” and “electrode placement.” Then, we clustered these co-occurring keywords. In general, we considered the clustering results reliable when the silhouette was > 0.7. Using the LLR clustering method, we obtained four clusters with silhouette > 0.7 and four clusters with silhouette > 0.8 (Table 5). In addition, the cluster with the largest size (0# magnetic resonance spectroscopy) has a contour value of 0.0661 (< 0.7), but we still consider the results of this cluster reliable. Based on co-occurring keywords and clustering, electroconvulsive therapy, treatment-resistant depression, bipolar disorder, hippocampus, efficacy, and electrode placement are popular research topics.

**TABLE 4:** Top 10 keywords in terms of frequency in the research.

Ranking	Keyword	Frequency
1	Electroconvulsive therapy	1,872
2	Major depression	851
3	Efficacy	397
4	Disorder	308
5	Depression	292
6	Meta analysis	259
7	Treatment resistant depression	238
8	Double blind	226
9	Bipolar disorder	141
10	Electrode placement	118

**TABLE 5:** Clusters of co-occurring keywords.

Cluster	Size	Silhouette	Mean year	Label (LLR algorithm)
0	75	0.661	2015	Magnetic resonance spectroscopy
1	73	0.761	2014	Propofol
2	73	0.805	2015	Hippocampus
3	60	0.760	2014	Electroconvulsive therapy
4	59	0.810	2014	Transcranial magnetic stimulation
5	43	0.748	2016	Machine learning
6	43	0.814	2016	Bipolar disorder
7	42	0.789	2014	Dementia
8	33	0.804	2016	Attitude

### Analysis of burst keywords and citation

**FIGURE 4:**
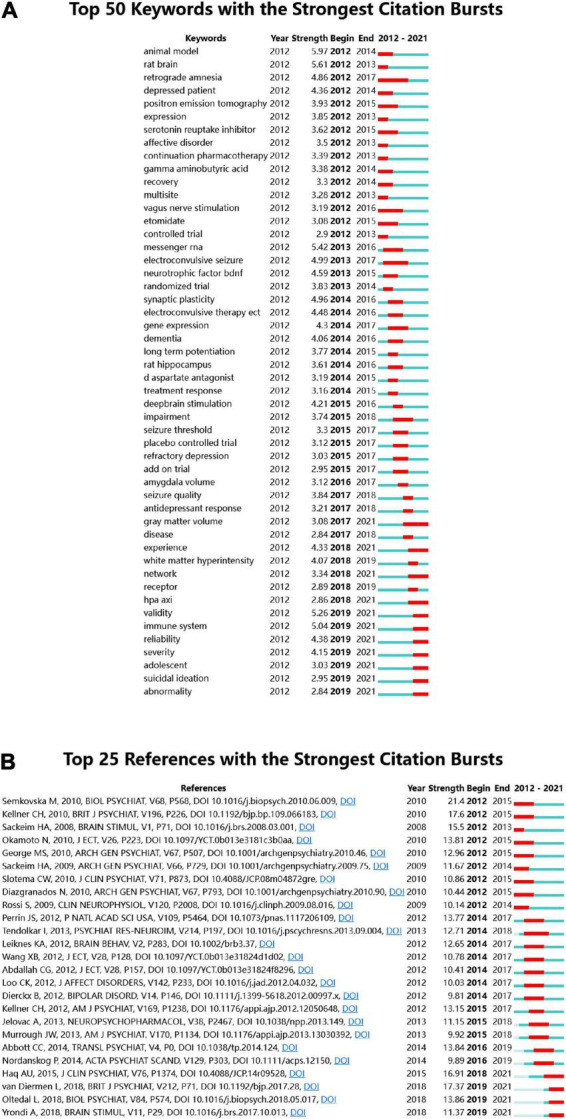
**(A)** Top 50 keywords with the strongest citation bursts. **(B)** Top 25 references with the strongest citation bursts.

The keyword bursts reflect hot topics and trends in the research field within a certain period of time. The blue line represents the last 10 years and the red line represents the duration of keyword bursts, demonstrating the evolution of hot topics. Figure 4 shows the 25 most cited keywords. “Retrograde amnesia” was the keyword with the longest duration in this field, lasting from 2006 to 2012, with a strength of 4.86. The keywords that last until 2021 mainly include “gray matter volume,” “network,” “adolescent,” and “suicidal ideation” (Figure 4A), which reflects the latest research trends.

Citation bursts of references can illustrate the evolution of the knowledge domain. Figure 4B lists the 25 references with high citation volumes. The duration of citation bursts is indicated by a red line segment. The most recent citation burst reference appeared in 2019 (van Diermen et al., 2018) with an intensity of 17.37. The strongest citation burst was a meta-analysis of ECT and cognitive function, published in 2010, with an intensity of 21.4. Three references had bursts that lasted until 2021 (Oltedal et al., 2018; van Diermen et al., 2018; Yrondi et al., 2018).

## Discussion

This study is the first to apply bibliometric and visual analysis methods to ECT and depressive disorder to visualize global hotspots and trends in ECT for depressive disorder over the past 10 years. The findings of this study may help researchers identify the changes in research hotspots in this field, help them select future research topics, and choose appropriate journals according to recent trends.

### Global trends in electroconvulsive therapy for depressive disorder

Our analysis showed that the number of publications in this field has been increasing since 2012. Among the many countries/regions with the highest number of publications, the United States has significantly contributed to the field, and most of the top 10 institutions in terms of number of publications are located in the United States. The University of Toronto, with 104 publications, is the institution with the highest number of publications in the field in the last decade, followed by Duke University and the University of California. Zafiris J. Daskalakis from the University of San Diego is the author with the highest number of publications, contributing to the field and collaborating with other researchers. It is noteworthy that China, as the third most published country, has made increasingly more research in this field in recent years. China also has collaborated with the US, the UK, and Australia. The prediction of research hotspots in the present study is expected to help Chinese researchers in their future research in this field and thus strengthen their international influence. One additional result emerging from the analysis is that Chongqing Medical University has published more papers in this field in the last decade, but has less cooperation with other institutions. International cooperation needs to be strengthened. We also found that the countries/regions or institutions that have contributed more to this field are located in Europe and the United States, while Southeast Asian or African countries have contributed less. We speculate that this may be related to the level of development and economic development of the country, which also indicates that the economic status of the country has an important influence on research development.

### Status of publications

This study found that most of the articles in this field were published in journals related to psychiatry, neurology, and behavioral medicine, and that the impact factors of the journals were high, which indicates that the distribution of articles was concentrated and of high quality. In addition, the top ten journals and the most cited journals are consistent, including many top journals, which also indicates the high quality of research and reliability of research results in this field. *The Journal of ECT* is the most published and most cited journal, and reports on clinical research and related projects related to ECT worldwide, covering all aspects of contemporary ECT and making a significant contribution to the field.

Among the ten most co-cited publications, seven were published in journals with impact factors higher than 10, and five were about changes in hippocampal volume after ECT. The most co-cited literature was published by Joshi et al. (2016) in *Biological Psychiatry*, who found that hippocampal volume was smaller in patients with depressive disorder (*p* < 0.04) and predicted clinical response after ECT (*p* < 0.05) by comparing hippocampal volume in patients with depressive disorder with controls at baseline. They also performed a longitudinal comparison of hippocampal and amygdala volumes in patients with depressive disorder before ECT, after two ECT sessions, and 1 week after completion of ECT and found significant increases in the volumes of the anterior hippocampus, basolateral, and centromedial amygdala. This suggests that ECT-induced neuroplasticity is associated with an elevated response and that these structural alterations may be related to neurotrophic processes. The results of some studies suggest an increase in brain-derived neurotrophic factor (BDNF) and other trophic factors after ECT, which is considered to be an important marker of cellular plasticity and these microscopic level changes may correspond to structural changes in imaging. Redlich et al. (2016) published in *JAMA Psychiatry* predicted the ECT treatment response of individuals based on pretreatment gray matter volume through machine learning. The prediction was 78.3% accurate (18/23) and 100% sensitive (13/13). The study also found that patients with depression with less damage to the structure of the subgenual cingulate cortex before ECT responded better to ECT. The results of this study serve as a catalyst for subsequent studies, providing a possible biological marker for individual responsiveness to ECT. The most recent and co-cited article is a meta-analysis by van Diermen et al. (2018) in the *British Journal of Psychiatry*, which is also a reference with the strongest citation burst lasting until 2021. This article provides a separate analysis of response and remission after ECT, and the results show that presence of psychotic features and older age can predict ECT remission and response, while severity of depression can only predict ECT response but not remission.

The above study explored the predictors of treatment response after ECT. The results suggest that both clinical features and neuroimaging findings are predictive of treatment response to ECT. In clinical practice, if we can identify patients with lower responsiveness to ECT early, we can choose other treatment options in the first place.

### Current research hotspots and frontiers in the future

We combined the cluster analysis of co-occurring words and bursts in terms of keywords and citations to predict future research hotspots in this field. In what follows, we examine the hotspots that emerged from our analysis, one by one.

#### Molecular biomarkers

The search for biological markers that can predict the response to treatment after ECT remains an important goal in the treatment of patients with depressive disorders. It has been shown that ECT has a neurotrophic effect, and it is likely that this effect is achieved by increasing the concentration of BDNF and vascular endothelial growth factor (VEGF) in the brain (Altar, 2004; Fournier and Duman, 2012; Polyakova et al., 2015).

Brain-derived neuro trophic factor is an indispensable neurotrophin in the development of the central nervous system, which not only promotes axonal growth and synaptic connections but also plays an important role in the mechanism of neuroplasticity (Machaalani and Chen, 2018). It has been demonstrated in animal models that electroconvulsive seizures (ECS, a recognized ECT analog in animal experiments) can elevate BDNF levels in the hippocampus while improving depression-like behavior (Zhang et al., 2016). However, it is inconclusive whether ECT is associated with elevated BDNF levels in studies of patients with depression. In fact, while Brunoni et al. (2014) showed that, similarly to the results of antidepressant treatment, peripheral BDNF levels are elevated in patients with depressive disorders after ECT, other scholars have come to the opposite conclusion (Polyakova et al., 2015). A recent study (Psomiades et al., 2022) suggested that this controversy might be due to selective measures of total BDNF levels in peripheral blood only. There are three subtypes of BDNF: BDNF precursor protein (proBDNF), mature BDNF (mBDNF), and BDNF prodomain (truncated). Both proBDNF and mBDNF are present in peripheral blood, but they play opposite roles. Since mBDNF facilitates neuronal cell plasticity (Bai et al., 2005), we speculate that mBDNF may play a greater role in the therapeutic effects of ECT than other isoforms. Psomiades et al. (2022) showed that higher baseline mBDNF levels are significantly associated with future remission after ECT. Thus, mBDNF is expected to be a predictor of ECT efficacy, and further studies are needed to test this hypothesis.

Vascular endothelial growth factor is a highly specific provascular endothelial growth factor that increases vascular endothelial permeability and is associated with processes such as neuronal growth and regeneration (Joon and Joseph, 2018). Animal experiments (Newton et al., 2003) have shown that VEGF levels in the rat hippocampus change after ECS, and may play an important role in the molecular action of ECS. With regard to studies on humans, it has been demonstrated that ECT increases serum VEGF levels in patients with refractory depressive disorder (Lucassen et al., 2001), and that low levels of VEGF may be associated with low responsiveness to ECT (Maffioletti et al., 2020). Elevated baseline VEGF levels have been shown to enhance the effects of ECT and increase drug concentrations in the brain (Minelli et al., 2014), which may be related to the transient disruption of the blood-brain barrier caused by ECT. Similarly, the reliability of VEGF as a predictor of ECT efficacy should be explored in the future.

#### Neuroimaging predictor

There is growing evidence that changes in hippocampal structure not only reflect the clinical state of major depressive disorder, but also correlate with treatment response. On the one hand, patients with depressive disorders have increased hippocampal apoptosis and reduced glial cell numbers (Sahay et al., 2007; Minelli et al., 2011); on the other, depressive episodes are associated with reduced hippocampal volume and abnormal functional connectivity of the hippocampus (Sheline et al., 1996; Masoud et al., 2013). Therefore, some scholars have suggested that the mechanism by which ECT works in depressive disorders is related to the enhancement of neuroplasticity and the restoration of hippocampal structure and function. As such, it has been hypothesized that hippocampal volume or its functional connectivity may be an important predictor of ECT efficacy. However, studies addressing this hypothesis to date provided mixed results. Some studies (Joshi et al., 2016; Xu et al., 2022) have shown that ECT selectively induces structural plasticity in the amygdala and hippocampal subregions associated with the antidepressant effects of ECT in patients with depressive disorder. It also seems to be associated with improved clinical responses. It has been hypothesized that an increase in hippocampal functional connectivity and volume may be biomarkers of ECT response, with smaller hippocampal volumes at baseline associated with stronger clinical responses (Abbott et al., 2014; Joshi et al., 2016). However, other studies suggest that the number of ECT sessions and electrode placement affect the degree of hippocampal enlargement, but that hippocampal volume changes do not correlate positively with clinical outcomes (Oltedal et al., 2018; Sartorius et al., 2019). As a result, it has been hypothesized that the efficacy of ECT cannot be explained by hippocampal enlargement alone. An increasing number of studies aim to explore imaging markers that can predict the efficacy of ECT; therefore, we speculate that this research direction is one of the future research hotspots.

#### Late-life depression

With the advent of China’s aging society (Zhao et al., 2014), an increasing number of elderly people are being diagnosed with depressive disorders, and ECT is considered to be an effective treatment for late-life depression (Kerner and Prudic, 2014). However, ECT can have negative effects on cognitive functions, especially on memory; therefore, the clinical use of ECT in the elderly population is more cautious. Recent studies (Kellner et al., 2016a) have suggested that ECT may not significantly exacerbate memory impairment and that the cognitive effects of ECT in elderly patients with depression are limited and transient (Kumar et al., 2016). In addition, a study in a *Lancet* subjournal suggested that ECT was not associated with the risk of incident dementia in patients with affective disorders (Osler et al., 2018), which provides additional evidence for the safety of ECT as a treatment option for depressive disorders in elderly people. It has also been suggested that the treatment effects of ECT may be better in older adults than in other age groups, but the opinion is inconclusive (Kellner et al., 2016b). A large-sample study (Luccarelli et al., 2022) from the real world, for instance, showed that the efficacy of ECT was independent of age. We speculate that the reason for the different results may be related to the source of the sample and the differences in the specific treatment parameters. The study also found that the older the age, the smaller the change in cognitive level after ECT treatment, but it only used the Montreal Cognitive Assessment as a cognitive assessment tool. Therefore, this finding needs to be verified through future studies collecting more and more reliable measures. The effect of ECT on cognitive function in elderly patients with depression remains unclear, and we believe that further studies will be conducted in this area in the future.

#### Adolescent and suicidal ideation

Adolescent suicide is one of the major mental health concerns at present. Some studies have shown that adolescent patients with major depressive disorder are prone to suicidal ideation as well as suicidal behavior (Patel et al., 2020). Epidemiological surveys have shown that the suicide rate among adolescent patients with depressive disorders in China is increasing year by year (Ma et al., 2019). In clinical practice, physicians use ECT as the first treatment option to alleviate severe suicidal ideation in adult patients with MDD, and it is also used in adolescent patients because its advantage of rapid onset and safety (UK ECT Review Group, 2003). However, some guardians of adolescent patients often refuse the treatment due to concerns about the cognitive impairments associated with ECT. Most studies have shown that ECT can cause transient impairments in cognitive function (e.g., immediate memory) (Semkovska and McLoughlin, 2010), but the impairments are reversible and can recover quickly after treatment is stopped (Semkovska and McLoughlin, 2010; Maric et al., 2016). Currently, there are fewer studies on ECT for suicidal ideation in adolescent patients with MDD, and studies on the efficacy and side effects of ECT for adolescent patients often involve multiple psychiatric disorders and lack comparability, so we believe that researchers will further explore this in the future in adolescent patients with depressive disorders with suicidal ideation.

### Limitations

This study has some limitations. First, our study only searched the WoSCC database. We believe this database is rich enough to provide an adequate base for the analysis, but conducting the same search on a different database might provide different results. Second, we only analyzed the results visually; therefore, it is possible that some publications were overlooked. Third, we only searched the literature in English and ignored publications in other languages. This approach may lead to overlook useful publications in languages other than English.

## Conclusion

This study is the first to apply bibliometric and visual analyses to an article on ECT for depressive disorder. We explored the current state of global research in the field and predicted future research trends. Since 2012, the number of publications has been increasing, with the United States and the University of Toronto being the most influential country and institution, respectively, and China as a relevant latecomer to the field. Daskalakis made a large contribution to the field as the author with the highest number of publications. *The Journal of ECT* is not only the most published journal, but also the most co-cited journal, and we predict that molecular biomarkers, neuroimaging predictors, and late-life depression will be relevant research hotspots in the future. The results of the present analysis of the literature suggest that more high-quality studies should be conducted to identify markers that can effectively predict ECT responsiveness. This would also help clinicians choose appropriate individualized treatment plans.

## Data availability statement

The raw data supporting the conclusions of this article will be made available by the authors, without undue reservation.

## Author contributions

HC and RD were responsible for study design and statistical analysis. ZW and KY were responsible for manuscript preparation. HC, RD, and WL were involved in evolving the ideas and edited the manuscript. All authors contributed to the article and approved the submitted version.

## References

[B1] Abbott C. C. Jones T. Lemke N. T. Gallegos P. McClintock S. M. Mayer A. R. (2014). Hippocampal structural and functional changes associated with electroconvulsive therapy response. *Trans. Psychiatry* 4:e483. 10.1038/tp.2014.124 25405780 PMC4259994

[B2] Altar C. (2004). Electroconvulsive seizures regulate gene expression of distinct neurotrophic signaling pathways. *J. Neurosci.* 24 2667–2677. 10.1523/JNEUROSCI.5377-03.2004 15028759 PMC6729526

[B3] Bai L. Pang P. T. Woo N. H. (2005). The yin and yang of neurotrophin action. *Nat. Rev. Neurosci.* 6 603–614. 10.1038/nrn1726 16062169

[B4] Brunoni A. R. Baeken C. Machado-Vieira R. Gattaz W. F. Vanderhasselt M. A. (2014). BDNF blood levels after electroconvulsive therapy in patients with mood disorders: A systematic review and meta-analysis. *World J. Biol. Psychiatry* 15 411–418. 10.3109/15622975.2014.892633 24628093

[B5] Chen C. Dubin R. Kim M. C. (2014). Emerging trends and new developments in regenerative medicine: A scientometric update (2000–2014). *Expert Opini. Biol. Ther.* 14:1295. 10.1517/14712598.2014.920813 25077605

[B6] Dukart J. Regen F. Kherif F. Colla M. Bajbouj M. Heuser I. (2014). Electroconvulsive therapy-induced brain plasticity determines therapeutic outcome in mood disorders. *Proc. Natl. Acad. Sci. U.S.A*. 111, 1156–1161. 10.1073/pnas.1321399111 24379394 PMC3903198

[B7] Fournier N. M. Duman R. S. (2012). Role of vascular endothelial growth factor in adult hippocampal neurogenesis: Implications for the pathophysiology and treatment of depression. *Behav. Brain Res.* 227 440–449. 10.1016/j.bbr.2011.04.022 21536078 PMC3176958

[B8] Haq A. U. Sitzmann A. F. Goldman M. L. Maixner D. F. Mickey B. J. (2015). Response of depression to electroconvulsive therapy: A meta-analysis of clinical predictors. *J. Clin. Psychiatry* 76 1374–1384. 10.4088/JCP.14r09528 26528644

[B9] Huang Y. Q. Wang Y. Wang H. Liu Z. Yu X. Yan J. (2019). Prevalence of mental disorders in China: A cross-sectional epidemiological study. *Lancet Psychiatry* 6 211–224. 10.1016/S2215-0366(18)30511-X 30792114

[B10] Jaffe R. (2002). *The practice of electroconvulsive therapy: Recommendations for treatment, training, and privileging: A task force report of the American psychiatric association*, 2nd Edn. Virginia: American Psychiatry Association. 10.1176/appi.ajp.159.2.331 41894194

[B11] Joon S. Joseph M. (2018). VEGF signaling in neurological disorders. *Int. J. Mol. Sci.* 19:275. 10.3390/ijms19010275 29342116 PMC5796221

[B12] Joshi S. H. Espinoza R. T. Pirnia T. Shi J. Wang Y. Ayers B. (2016). Structural plasticity of the hippocampus and amygdala induced by electroconvulsive therapy in major depression. *Biol. Psychiatry* 79 282–292. 10.1016/j.biopsych.2015.02.029 25842202 PMC4561035

[B13] Kellner C. H. Husain M. M. Knapp R. G. McCall W. V. Petrides G. Rudorfer M. V. (2016a). A novel strategy for continuation ect in geriatric depression: Phase 2 of the PRIDE study. *Am. J. Psychiatry* 173 1110–1118. 10.1176/appi.ajp.2016.16010118 27418381 PMC7130448

[B14] Kellner C. H. Husain M. M. Knapp R. G. McCall W. V. Petrides G. Rudorfer M. V. (2016b). Right unilateral ultrabrief pulse ECT in geriatric depression: Phase 1 of the PRIDE study. *Am. J. Psychiatry* 173 1101–1109. 10.1176/appi.ajp.2016.15081101 27418379 PMC7130447

[B15] Kerner N. Prudic J. (2014). Current electroconvulsive therapy practice and research in the geriatric population. *Neuropsychiatry* 4 33–54. 10.2217/npy.14.3 24778709 PMC4000084

[B16] Kumar S. Mulsant B. H. Liu A. Y. Blumberger D. M. Daskalakis Z. J. Rajji T. K. (2016). Systematic review of cognitive effects of electroconvulsive therapy in late-life depression. *Am. J. Geriatr. Psychiatry* 24 547–565. 10.1016/j.jagp.2016.02.053 27067067

[B17] Lucassen P. J. Müller M. B. Holsboer F. Bauer J. Holtrop A. Wouda J. (2001). Hippocampal apoptosis in major depression in a minor event and absent from subareas at risk for glucocorticoid overexposure. *Am. J. Pathol.* 158 453–468. 10.1016/S0002-9440(10)63988-0 11159183 PMC1850286

[B18] Luccarelli J. McCoy T. H. Seiner S. J. Henry M. E. (2022). Real-world evidence of age-independent electroconvulsive therapy efficacy: A retrospective cohort study. *Acta Psychiatr. Scand.* 145 100–108. 10.1111/acps.13378 34662429 PMC8709695

[B19] Ma Y. J. Wang D. F. Yuan M. Zhang X. J. Long J. Chen S. B. (2019). The prevalence, metabolic disturbances and clinical correlates of recent suicide attempts in Chinese inpatients with major depressive disorder. *BMC Psychiatry* 19:144. 10.1186/s12888-019-2131-6 31077181 PMC6509770

[B20] Machaalani R. Chen H. (2018). Brain derived neurotrophic factor (BDNF), its tyrosine kinase receptor B (TrkB) and nicotine. *Neurotoxicology* 65 186–195. 10.1016/j.neuro.2018.02.014 29499216

[B21] Maffioletti E. Gennarelli M. Magri C. Bocchio-Chiavetto L. Bortolomasi M. Bonvicini C. (2020). Genetic determinants of circulating VEGF levels in major depressive disorder and electroconvulsive therapy response. *Drug Dev. Res.* 81 593–599. 10.1002/ddr.21658 32173896

[B22] Maric N. P. Stojanovic Z. Andric S. Soldatovic I. Dolic M. Spiric Z. (2016). The acute and medium-term effects of treatment with electroconvulsive therapy on memory in patients with major depressive disorder. *Psychol. Med.* 46 797–806. 10.1017/S0033291715002287 26493090

[B23] Masoud T. Knight D. C. Manoliu A. Schwerthöffer D. Scherr M. Meng C. (2013). Aberrant intrinsic connectivity of hippocampus and amygdala overlap in the fronto-insular and dorsomedial-prefrontal cortex in major depressive disorder. *Front. Hum. Neurosci.* 7:639. 10.3389/fnhum.2013.00639 24101900 PMC3787329

[B24] Minelli A. Maffioletti E. Bortolomasi M. Conca A. Zanardini R. Rillosi L. (2014). Association between baseline serum vascular endothelial growth factor levels and response to electroconvulsive therapy. *Acta Psychiatr. Scand.* 129 461–466. 10.1111/acps.12187 23957507

[B25] Minelli A. Zanardini R. Abate M. Bortolomasi M. Gennarelli M. Bocchio-Chiavetto L. (2011). Vascular endothelial growth factor (VEGF) serum concentration during electroconvulsive therapy (ECT) in treatment resistant depressed patients. *Prog. Neuropsychopharmacol. Biol. Psychiatry* 35 1322–1325. 10.1016/j.pnpbp.2011.04.013 21570438

[B26] National Institute for Clinical Excellence [NICE] (2003). *Guidance on the use of electroconvulsive therapy.* London: National Institute for Clinical Excellence.

[B27] Newton S. S. Collier E. F. Hunsberger J. Adams D. Terwilliger R. Selvanayagam E. (2003). Gene profile of electroconvulsive seizures: Induction of neurotrophic and angiogenic factors. *J. Neurosci.* 23 10841–10851. 10.1523/jneurosci.23-34-10841.2003 14645477 PMC6740983

[B28] Oltedal L. Narr K. L. Abbott C. Anand A. Argyelan M. Bartsch H. (2018). Volume of the human hippocampus and clinical response following electroconvulsive therapy. *Biol. Psychiatry* 84 574–581. 10.1016/j.biopsych.2018.05.017 30006199 PMC6697556

[B29] Osler M. Rozing M. P. Christensen G. T. Andersen P. K. Jørgensen M. B. (2018). Electroconvulsive therapy and risk of dementia in patients with affective disorders: A cohort study. *Lancet Psychiatry* 5 348–356. 10.1016/S2215-0366(18)30056-7 29523431

[B30] Patel R. S. Onyeaka H. Youssef N. A. (2020). Suicidal ideation and attempts in unipolar versus bipolar depression: Analysis of 131,740 adolescent inpatients nationwide. *Psychiatr. Res.* 291:113231. 10.1016/j.psychres.2020.113231 32574899

[B31] Polyakova M. Schroeter M. L. Elzinga B. M. Holiga S. Schoenknecht P. de Kloet E. R. (2015). Brain-derived neurotrophic factor and antidepressive effect of electroconvulsive therapy: Systematic review and meta-analyses of the preclinical and clinical literature. *PLoS One* 10:e0141564. 10.1371/journal.pone.0141564 26529101 PMC4631320

[B32] Psomiades M. Mondino M. Galvão F. Mandairon N. Nourredine M. Suaud-Chagny M. F. (2022). Serum mature BDNF level is associated with remission following ECT in treatment-resistant depression. *Brain Sci.* 12:126. 10.3390/brainsci12020126 35203890 PMC8870188

[B33] Ravindran A. V. Lam R. W. Filteau M. J. Lespérance F. Kennedy S. H. Parikh S. V. (2009). Canadian network for mood and anxiety treatments (CANMAT) clinical guidelines for the management of major depressive disorder in adults. *J. Affect. Disord.* 117(Suppl. 1) S54–S64. 19666194 10.1016/j.jad.2009.06.040

[B34] Redlich R. Opel N. Grotegerd D. Dohm K. Zaremba D. Bürger C. (2016). Prediction of individual response to electroconvulsive therapy via machine learning on structural magnetic resonance imaging data. *JAMA Psychiatry* 73:557. 10.1001/jamapsychiatry.2016.0316 27145449

[B35] Rush A. J. Trivedi M. H. Wisniewski S. R. Nierenberg A. A. Stewart J. W. Warden D. (2006). Acute and longer-term outcomes in depressed outpatients requiring one or several treatment steps: A STAR*D report. *Am. J. Psychiatry* 163 1905–1917. 10.1176/ajp.2006.163.11.1905 17074942

[B36] Sahay A. Drew M. R. Hen R. (2007). Dentate gyrus neurogenesis and depression. *Prog. Brain Res.* 163 697–722. 10.1016/S0079-6123(07)63038-6 17765746

[B37] Sartorius A. Demirakca T. Böhringer A. Clemm von Hohenberg C. Aksay S. S. Bumb J. M. (2019). Electroconvulsive therapy induced gray matter increase is not necessarily correlated with clinical data in depressed patients. *Brain Stimul.* 12 335–343. 10.1016/j.brs.2018.11.017 30554869

[B38] Semkovska M. Landau S. Dunne R. Kolshus E. Kavanagh A. Jelovac A. (2016). Bitemporal versus high-dose unilateral twice-weekly electroconvulsive therapy for depression (EFFECT-Dep): A pragmatic, randomized, non-inferiority trial. *Am. J. Psychiatry* 173, 408–417. 10.1176/appi.ajp.2015.15030372 26892939

[B39] Semkovska M. McLoughlin D. M. (2010). Objective cognitive performance associated with electroconvulsive therapy for depression: A systematic review and meta-analysis. *Biol. Psychiatry* 68 568–577. 10.1016/j.biopsych.2010.06.009 20673880

[B40] Sheline Y. I. Wang P. W. Gado M. H. Csernansky J. G. Vannier M. W. (1996). Hippocampal atrophy in recurrent major depression. *Proc. Natl. Acad. Sci. U.S.A.* 93 3908–3913. 10.1073/pnas.93.9.3908 8632988 PMC39458

[B41] Tor P. C. Tan X. W. Martin D. Loo C. (2021). Comparative outcomes in electroconvulsive therapy (ECT): A naturalistic comparison between outcomes in psychosis, mania, depression, psychotic depression and catatonia. *Eur. Neuropsychopharmacol.* 51 43–54. 10.1016/j.euroneuro.2021.04.023 34034099

[B42] UK ECT Review Group (2003). Efficacy and safety of electroconvulsive therapy in depressive disorders: A systematic review and meta-analysis. *Lancet* 361 799–808. 10.1016/S0140-6736(03)12705-5 12642045

[B43] van Diermen L. van den Ameele S. Kamperman A. M. Sabbe B. C. G. Vermeulen T. Schrijvers D. (2018). Prediction of electroconvulsive therapy response and remission in major depression: Meta-analysis. *Br. J. Psychiatry* 212 71–80. 10.1192/bjp.2017.28 29436330

[B44] Wang H. Tian X. Wang X. Wang Y. (2021). Evolution and emerging trends in depression research from 2004 to 2019: A literature visualization analysis. *Front. Psychiatry* 12:705749. 10.3389/fpsyt.2021.705749 34777037 PMC8585938

[B45] World Health Organization [WHO] (2020). *Depression.* Geneva: World Health Organization.

[B46] Xu J. Li W. Bai T. Li J. Zhang J. Hu Q. (2022). Volume of hippocampus-amygdala transition area predicts outcomes of electroconvulsive therapy in major depressive disorder: High accuracy validated in two independent cohorts. *Psychol. Med.* 1–10. 10.1017/S0033291722001337 35604047

[B47] Yrondi A. Sporer M. Péran P. Schmitt L. Arbus C. Sauvaget A. (2018). Electroconvulsive therapy, depression, the immune system and inflammation: A systematic review. *Brain Stimul.* 11 29–51. 10.1016/j.brs.2017.10.013 29111078

[B48] Zhang F. Luo J. Min S. Ren L. Qin P. (2016). Propofol alleviates electroconvulsive shock-induced memory impairment by modulating proBDNF/mBDNF ratio in depressive rats. *Brain Res.* 1642 43–50. 2016.03.020 10.1016/j.brainres.2016.03.020 27017958

[B49] Zhao Y. Hu Y. Smith J. P. Strauss J. Yang G. (2014). Cohort profile: The China health and retirement longitudinal study (CHARLS). *Int. J. Epidemiol.* 43 61–68. 10.1093/ije/dys203 23243115 PMC3937970

